# Investigation and Modeling of Multi-Node Body Channel Wireless Power Transfer †

**DOI:** 10.3390/s20010156

**Published:** 2019-12-25

**Authors:** Yuxuan Huang, Jian Zhao, Wenyu Sun, Huazhong Yang, Yongpan Liu

**Affiliations:** 1 Department of Electronic Engineering and Beijing Innovation Center for Future Chips, Tsinghua University, Beijing 100084, China; hyx17@mails.tsinghua.edu.cn (Y.H.); wy-sun16@mails.tsinghua.edu.cn (W.S.); yanghz@tsinghua.edu.cn (H.Y.); 2 Department of Micro/Nano Electronics, Shanghai Jiao Tong University, Shanghai 200240, China

**Keywords:** body channel wireless power transfer (BC-WPT), empirical circuit model, inter-degeneration mechanism, theoretical circuit analysis, multiple nodes

## Abstract

Insufficient power supply is a huge challenge for wireless body area network (WBAN). Body channel wireless power transfer (BC-WPT) is promising to realize multi-node high-efficiency power transmission for miniaturized WBAN nodes. However, the behavior of BC-WPT, especially in the multi-node scenario, is still lacking in research. In this paper, the inter-degeneration mechanism of a multi-node BC-WPT is investigated based on the intuitive analysis of the existing circuit model. Co-simulation in the Computer Simulation Technology (CST) and Cadence platform and experiments in a general indoor environment verify this mechanism. Three key factors, including the distance between the source and the harvester, frequency of the source, and area of the ground electrodes, are taken into consideration, resulting in 15 representative cases for simulation and experiments studies. Based on the simulation parameters, an empirical circuit model to accurately predict the received power of multiple harvesters is established, which fits well with the measurement results, and can further provide guidelines for designs and research on multi-node BC-WPT systems.

## 1. Introduction

**Figure 1:**
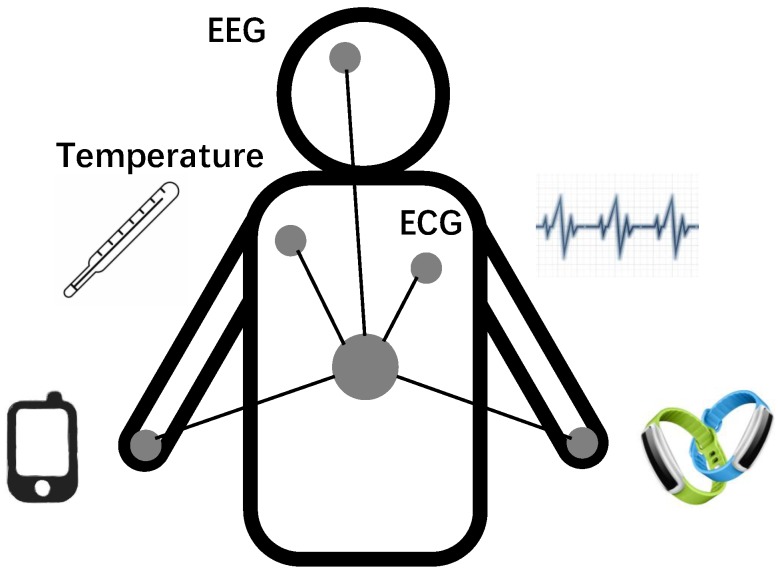
Illustration of wireless body area network (WBAN) application. EEG = electroencephalograph; ECG = electrocardiograph.

Today, more and more wearable devices are appearing, which collect health information, such as electrocardiograph (ECG), electroencephalograph (EEG), electromyogram (EMG), blood pressure, body temperature, and other vital signals, for utilization in health monitoring and medical diagnosis through a wireless body area network (WBAN), as shown in Figure 1 [1]. These kinds of wearable biomedical sensor nodes are battery-powered in the majority of instances, and the battery needs to be charged or replaced periodically. In addition, the human posture may affect the stability of a WBAN and needs to be estimated [2]. To meet the requirements of WBAN characteristics, smaller node size has become the optimization target of sensor nodes, which results in lower battery capacity and more frequent battery charging or replacement. As a consequence, the design of WBAN nodes encounters the dilemma between lifetime and volume.

To solve this problem, wireless power transfer (WPT) is one approach for battery-powered WBAN. Several researchers have utilized different methods to optimize WPT efficiency. In [3], the inductive coupling was studied with detailed theoretical and numerical analysis. In [4], the magnetic resonance coupling using self-resonant coils in a strongly coupled regime was first proposed. Ref. [5] studied RF radiation with nondirective far-field powering at higher frequencies with antennas. The above three methods are the most common traditional WPT technologies. However, coils and antennas are required in these technologies, which increase the size and thus are not suitable for miniaturized nodes. Besides, RF radiation may be harmful to the human body when the exposure density is high according to the IEEE Standard for Safety Levels [6] and the Federal Communications Commission (FCC) Rules [7].

Body channel communication (BCC) technology is a new method to transmit data included in IEEE Standard 802.15.6 [8]. Several researches have focused on the characteristics and modeling of BCC. [9] used finite-element method to study the channel characteristics of BCC. In [10], the skin propagation model was proposed based on a distributed-parameter circuit. In [11], the body transmission channel model was proposed based on RC distribution networks and transmission line theory. In [12], the human head and neck model was proposed. Today, there are already some mature applications using BCC technology. NTT Corporation has developed Firmo evaluation kit using BCC technology [13]. Ad-Sol Nissin Corporation has also launched the wireless touch tag reader using human body as communication channel [14]. In [15], it is also mentioned that health data can be exchanged with a Wearable Hub through BCC network.

Due to the advantages of low transmission loss and no need for large antennas studied in the above researches, BCC can be also used in power transmission through WBAN nodes. In [16], a BCC identification system with $8.88\times 10^{-3}$ W/W power transmission efficiency was first published, which delivers energy through the human body channel without carefully aligned coils or antennas. In [17], the inter-degeneration effect of this energy transmission method is investigated. However, theoretical analysis is missing in the above-mentioned body channel WPT (BC-WPT) technology and it only discusses the single-source single-harvester case. With increasing sensor nodes for health monitoring and medical diagnosis, multi-node BC-WPT needs to be seriously considered and investigated, which is still lack of research now.

In this work, the behavior of one source (S) and multiple harvesters (H) in BC-WPT has been studied for the first time. To provide a theoretical basis for simulation and experiments, the behavior analysis of BC-WPT is carried out. The results of the behavior analysis give the key factors affecting BC-WPT and also give preliminary trend results. Based on CST and Cadence co-simulation platform, the multi-node BC-WPT is studied and the S-parameter model is simulated. The simulation results not only give the circuit parameters of the proposed empirical circuit model but also investigates the inter-degeneration mechanism between multi-node received power. Three key factors including the distance between the source and the harvester, frequency of the source, and area of the ground electrodes, are simulated in 15 cases. To verify the characteristics of a multi-node BC-WPT, practical experiments are carried out for cross-validation with simulation. Based on the simulation parameters, an empirical circuit model of the received power of multiple harvesters in multi-node BC-WPT is established, which can fit the measurement results well.

The contributions and advantages of the proposed multi-node modeling of BC-WPT are listed as follows.

- The behavior of the multi-node BC-WPT is first analyzed to provide a theoretical basis for simulation and experiments. Three key factors are given to provide guidelines for the selection of cases in simulation.
- The inter-degeneration mechanism between multiple nodes is investigated. According to the key factors based on the theoretical analysis, 15 cases are considered. Simulation and experiments are carried out to verify the impact of these key factors on inter-degeneration mechanism.
- The empirical circuit model of the multi-node BC-WPT is first proposed. The circuit parameters are extracted from the co-simulation platform. The simulation S-parameter model and practical experiments are carried out for verification.

The rest of this paper is organized as follows. Section 2 introduces the overall BC-WPT system and the theoretical analysis of its behavior. Section 3 describes the simulation platform and different simulation cases of the multi-node BC-WPT. Section 4 illustrates the experiment setup of BC-WPT and analyzes the measurement and simulation results. An empirical circuit model is established in Section 5 which can predict the transmission power of BC-WPT with tolerable errors. Finally, Section 6 concludes this paper.

## 2. Overview and Behavior Analysis of BC-WPT

### 2.1. Overview of BC-WPT

BCC is a communication technology, which can be classified into galvanic coupling BCC (GC-BCC) and capacitive coupling BCC (CC-BCC) according to the transmission scheme [18]. CC-BCC is safer than GC-BCC because there is a large transmission current in GC-BCC, which may be harmful to human body [19,20]. In CC-BCC, the forward path is constituted by the human body between the signal electrodes of the transmitter and the receiver, while the backward path is composed of the coupling between ground electrodes of the transmitter and the receiver. Thanks to the high conductivity of the human body [21] and no need for large-size coils or antennas, BCC has the advantages of lower transmission loss and miniaturization.

**Figure 2:**
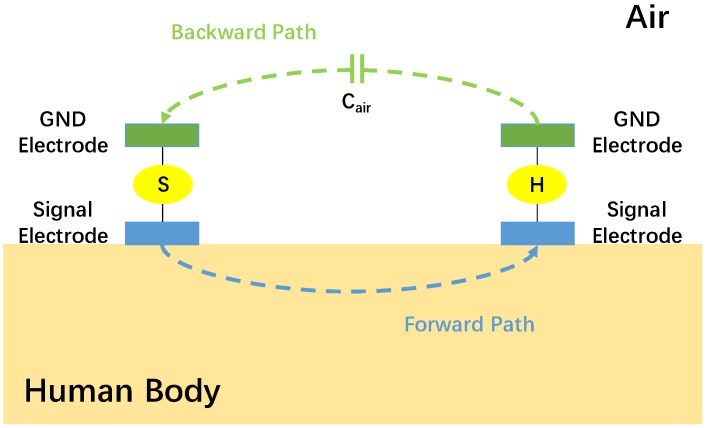
System block diagram of body channel wireless power transfer (BC-WPT) [17]. GND = ground.

WPT is an effective approach to remotely charge the battery for the WBAN nodes. Traditional WPT solves the problem of frequent battery charging or replacement, while it brings new problems of large coils or antennas. In addition, the coils need to be aligned and cannot support multi-node power transfer. However, BC-WPT is a new power transmission technology which takes the advantages of BCC. The E-fields around human body, especially at the extremities of the body, are much stronger than that in the air, which makes BC-WPT possible [22]. The signal electrodes are attached to the human body, while the ground electrodes are suspended, as shown in Figure 2. Compared with traditional WPT technology, the transmission distance is longer and the transmission loss is smaller. No need for coils alignment makes BC-WPT support simultaneous multi-node power transfer, more convenient application, and low-power systems [23,24].

In BC-WPT, the single-source single-harvester (SSSH) case and the single-source multi-harvester (SSMH) case are considered in this work. The SSSH case means there are one source and one harvester deployed on the human body, while the SSMH case refers to one source and multiple harvesters arranged on the human body. In particular, the SSMH case can be divided into the SSMH-SA case and SSMH-OP case according to the location of multiple harvesters, where the SSMH-SA case means multiple harvesters are on the same side of the source, while the SSMH-OP case means multiple harvesters are on the opposite side of the source.

### 2.2. Behavior Analysis of SSSH BC-WPT

**Figure 3:**
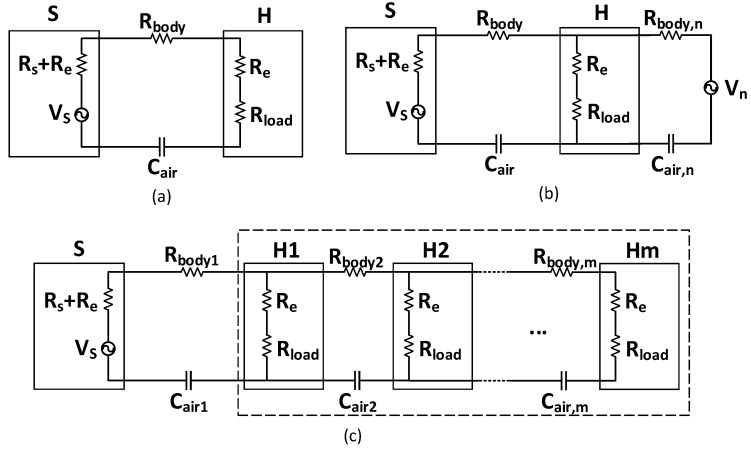
The simplified equivalent circuit model of BC-WPT. (**a**) The single-source single-harvester (SSSH) case. (**b**) The SSSH case with noise. (**c**) The multi-node case where harvesters are on the same side of the source.

To intuitively study the characteristic of the multi-node BC-WPT, the simplified equivalent circuit model is used for analysis [25]. As the frequency of the signal is tens of MHz whose wavelength is much larger than the human body size, the electric field can be regarded as a quasi-static field and the human body channel can be replaced by lumped resistance and capacitance. As shown in Figure 3a in the SSSH case, the forward path is equivalent to $R_{body}$ and the backward path is equivalent to $C_{air}$, which is related to the distance (*D*) between the source and the harvester. The electrode resistance is $R_{e}$ and the resistance of the source is $R_{s}$. The voltage of the source is $V_{s}$ and the load of the harvester is $R_{load}$. As a result, the received voltage on resistance load $V_{load}$ is obtained as

(1) $$V_{load}=\frac{R_{load}V_{s}}{R_{s}+R_{load}+2R_{e}+R_{body}\left(D\right)+\frac{1}{j\omega C_{air}\left(D\right)}}.$$

When the distance between the source and the harvester becomes longer, $R_{body}$ becomes larger and $C_{air}$ becomes smaller, leading to lower voltage magnitude $|V_{load}|$.

The difference between WPT and wireless communication is that WPT is concerned with power efficiency, while wireless communication is more concerned with signal noise ratio (SNR) and sensitivity. As shown in Figure 3b, if a noise source $V_{n}$ is added to the system in SSSH case, the square of the received voltage on the harvester $|V_{load,h}{|}^{2}$ is obtained as

(2) $$|V_{load,h}{|}^{2}={\left|\frac{Z_{eq1}V_{s}}{R_{s}+R_{e}+R_{body}+\frac{1}{j\omega C_{air}}+Z_{eq1}}+\frac{Z_{eq2}V_{n}}{R_{body,n}+\frac{1}{j\omega C_{air,n}}+Z_{eq2}}\right|}^{2},$$

where

(3) $$\begin{array}{cc} \; & Z_{eq1}=\left(R_{e}+R_{load}\right)\left|\right|\left(R_{body,n}+\frac{1}{j\omega C_{air,n}}\right),\\ \; & Z_{eq2}=\left(R_{e}+R_{load}\right)\left|\right|\left(R_{s}+R_{e}+R_{body}+\frac{1}{j\omega C_{air}}\right). \end{array}$$

$R_{body,n}$ and $C_{air,n}$ are the forward path and the backward path of the noise source respectively. As a result, the noise will reduce the SNR of the signal in wireless communication. If the noise power doubles, the SNR will decrease by 3 dB. Thus the bit error ratio (BER) will increase and it is hard to demodulate the original signal [26]. However, SNR is not the key performance in BC-WPT, so there is no need to consider this issue.

### 2.3. Behavior Analysis of SSMH BC-WPT and Inter-Degeneration Mechanism

The multi-node case is quite different from the aforementioned SSSH case as multiple nodes will change the electric field distribution. As a result, the power transmission paths will be no longer independent of each other. As shown in Figure 3c in the multi-node case, taking the case where the harvesters are on the same side of the source as an example, equivalent resistance $R_{eq}$ in the dotted frame is calculated, whose recursive equation can be written as

(4) $$\begin{array}{cc} \; & R_{eq,m}=R_{eq,m-1}\left|\right|\left(R_{e}+R_{load}+R_{body,m}+\frac{1}{j\omega C_{air,m}}\right),\\ \; & R_{eq,1}=R_{e}+R_{load}, \end{array}$$

where *m* is the number of the harvesters, and $R_{eq,m}$ represents the equivalent load resistance when the number of harvesters is *m*. Let’s consider the SSMH-SA case specifically where *m* is equal to 2. Let $R_{1}=R_{e}+R_{load}$, $R_{2}=R_{1}+R_{body2}$, $C=C_{air2}$, and the equivalent resistance $R_{eq,2}$ is obtained as

(5) $$R_{eq,2}=\frac{R_{e}+R_{load}}{1+A+j\omega B},$$

where

(6) $$A=\frac{\omega^{2}R_{1}R_{2}C^{2}}{1+\omega^{2}{R_{2}}^{2}C^{2}},B=\frac{R_{1}C}{1+\omega^{2}{R_{2}}^{2}C^{2}}.$$

Since *A* is greater than 0, the resistance magnitude $|R_{eq,2}|$ is always smaller than $R_{e}+R_{load}$, which means the second harvester has the degeneration mechanism on the first harvester. Considering ωB/(1+A), it can be yielded as

(7) $$\frac{\omega B}{1+A}=\frac{\omega R_{1}C}{1+\omega^{2}\left(R_{1}R_{2}+{R_{2}}^{2}\right)C^{2}}\leq \frac{R_{1}}{2\sqrt{R_{1}R_{2}+{R_{2}}^{2}}}.$$

Since $R_{1}$ is always smaller than $R_{2}$, $max\left\{\omega B/\left(1+A\right)\right\}=1/2\sqrt{2}$, and the maximum error between the real part and the magnitude is $(3-2\sqrt{2})/3=5.72\%$. As a result, the imaginary part of jωB can be ignored and the magnitude is approximately equal to the real part. Since $R_{body2}$ is proportional to the distance and $C_{air2}$ is inversely proportional to the distance *L* [20,27], let $R_{2}=R_{1}+\alpha L$ and C=β/L, and *A* can be expressed as

(8) $$A=\frac{\beta^{2}\omega^{2}R_{1}\left(R_{1}+\alpha L\right)}{L^{2}+\beta^{2}\omega^{2}{\left(R_{1}+\alpha L\right)}^{2}}.$$

When the distance between two harvesters becomes shorter, *A* becomes larger and $R_{eq,2}$ becomes smaller, which means the inter-degeneration mechanism becomes stronger and the received signal strength becomes lower. Extremely, when *L* tends to be zero, $R_{eq,2}$ tends to be $(R_{e}+R_{load})/2$ and the inter-degeneration mechanism reaches the most significant point. When *L* tends to be positive infinity, the SSMH-SA case changes into the SSSH case and the inter-degeneration mechanism disappears. As shown in the above equations, ω, *L*, $R_{body}$, and $C_{air}$ are the key factors in BC-WPT and these key factors will be discussed in detail in the next sections.

## 3. Simulation Setup of BC-WPT

In this section, simulation setup of the multi-node BC-WPT is illustrated. The first subsection displays the constructed S-parameter model of the human body in CST. The second subsection shows the CST and Cadence co-simulation platform. Several simulation cases are given in the third subsection.

### 3.1. S-Parameter Model in CST

**Figure 4:**
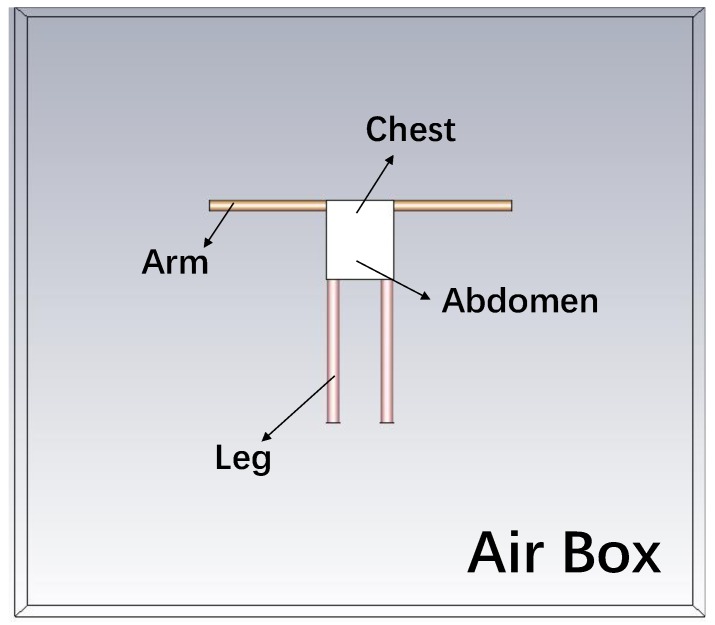
Simulation platform in Computer Simulation Technology (CST) [17].

CST tool is used to construct the S-parameter model of the human body in this simulation. The simulation platform mimics the human shape of standing posture and consists of two arms, two legs, one abdomen, and one chest in the air box, as shown in Figure 4. Five tissue layers make up the arm and leg models, which include skin, fat, muscle, cortical bone, and bone marrow, while four tissue layers form the abdomen and chest models which include skin, fat, muscle, and organs. Take the arm as an example to discuss the shape of human body. In [22], the arm is modeled as cuboid. However, in most of the cases, the arm is modeled as cylinder [9,28]. Since the shape of the arm is more similar to a cylinder than a cuboid in reality, we use cylinder to model the arm. The thickness of each tissue layer is chosen in Table 1 according to [28]. The S-parameter of the body channel can be obtained by simulation in CST and construct the S-parameter model.

### 3.2. Co-Simulation Platform

**Figure 5:**
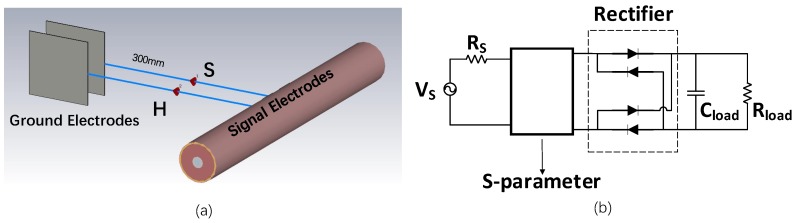
Co-simulation platform taking the SSSH case as an example. (**a**) Setup in CST. (**b**) Complete simulation circuits in Cadence [17].

The co-simulation platform is shown in Figure 5. An arm model is used for simulation and experiments. In CST, as shown in Figure 5a, the distance between the signal electrode and the ground electrode is fixed to 300 mm and the discrete port is located between them. To be consistent with the experiment setup in Section 4, the impedance of the source port is chosen to be 50 Ω, while that of the harvester port is chosen to be 2 kΩ. The area of the signal electrodes is fixed to 30 mm× 30 mm, while that of the ground electrodes is not fixed and used as simulation cases.

The complete simulation circuit in Cadence is shown in Figure 5b. The power source is a sinusoidal wave with upper and lower peak voltages of 3 V and 0 V, respectively, whose resistance is 50 Ω. The S-parameter of the discrete ports of human body channel is extracted from CST. A bridge rectifier is adopted with four diodes, a 2 kΩ load and a 100 pF capacitor in parallel. The highest transmission power is 10.51 dBm, which is within the human safety range [29].

### 3.3. Simulation Cases of S-Parameter Model

There is a little previous work in BC-WPT. [16] displayed power transfer through human body. However, the paper did not focus on the factors which affect power transfer and only showed the SSSH case. In addition, it had no simulation in the paper. [17] investigated the characteristics of BC-WPT. However, the paper only focused on the factor of distance between source and harvester.

In this paper, according to the conclusion drawn in Section 2 that ω, *L*, $R_{body}$, and $C_{air}$ are the key factors in BC-WPT, three general simulation cases are discussed in both SSSH and SSMH cases in this subsection. Case I, II, and III study the relationship between received power and distance between the source and the harvester, frequency of the source and area of the ground electrodes, respectively, as shown in Table 2. Two harvesters are used as examples in SSMH cases.

#### 3.3.1. Cases of Distance Between S and H (Case I)

**Figure 6:**
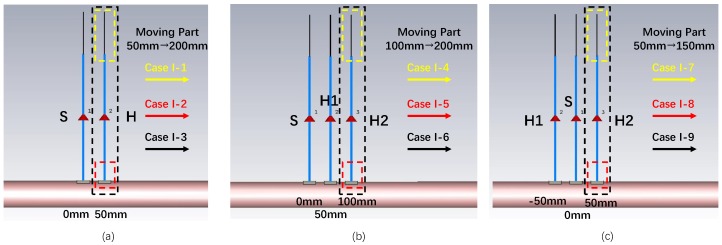
Simulation Case I. (**a**) Case I-1∼Case I-3: The SSSH cases. In Case I-1, forward distance is fixed and backward distance changes. In Case I-2, backward distance is fixed and forward distance changes. In Case I-3, forward and backward distances are the same. (**b**) Case I-4∼Case I-6: The SMH-SA cases where S and H1 are fixed. In Case I-4, the signal electrode of H2 is fixed, and the ground electrode of H2 changes. In Case I-5, the ground electrode of H2 is fixed and signal electrode of H2 changes. In Case I-6, the position of signal and ground electrodes of H2 is the same. (**c**) Case I-7∼Case I-9: The SSMH-OP cases where S and H1 are fixed. In Case I-7, the signal electrode of H2 is fixed, and the ground electrode of H2 changes. In Case I-8, ground electrode of H2 is fixed, and the signal electrode of H2 changes. In Case I-9, the position of signal and ground electrodes of H2 is the same.

When discussing Case I, the frequency of the source is fixed to 20 MHz and the area of the ground electrodes is fixed to 100 mm× 100 mm. Figure 6 shows nine simulation cases in Case I, where different distances between S and H are simulated. Case I-1∼Case I-3 are the SSSH cases. In Case I-1, the forward distance between S and H is fixed to 50 mm and the backward distance changes from 50 mm to 200 mm at 10 mm per step. In Case I-2, the backward distance between S and H is fixed to 50 mm and the forward distance changes from 50 mm to 200 mm at 10 mm per step. In Case I-3, the distances of the forward path and the backward path are the same and the distance between S and H changes from 50 mm to 200 mm at 10 mm per step. Case I-4∼Case I-6 are the SSMH-SA cases, where S and harvester 1 (H1) are fixed in 0 mm and 50 mm position. In Case I-4, the position of the signal electrode of harvester 2 (H2) is fixed to 100 mm and that of the ground electrode of H2 changes from 100 mm to 200 mm at 10 mm per step. In Case I-5, the position of the ground electrode of H2 is fixed to 100 mm and that of the signal electrode of H2 changes from 100 mm to 200 mm at 10 mm per step. In Case I-6, the position of the signal electrode and the ground electrode is the same and changes from 100 mm to 200 mm at 10 mm per step. Case I-7∼Case I-9 are the SSMH-OP cases, where S and H1 are fixed in 0 mm and −50 mm position. In Case I-7, the position of the signal electrode of H2 is fixed to 50 mm and that of the ground electrode of H2 changes from 50 mm to 150 mm at 10 mm per step. In Case I-8, the position of the ground electrode of H2 is fixed to 50 mm and that of the signal electrode of H2 changes from 50 mm to 150 mm at 10 mm per step. In Case I-9, the position of the signal electrode and the ground electrode is the same and changes from 50 mm to 150 mm at 10 mm per step.

#### 3.3.2. Cases of Frequency of the Source (Case II)

When discussing Case II, the area of the ground electrodes is fixed to 100 mm× 100 mm. The distances of the forward path and the backward path are the same. Nine different frequencies, including 20 MHz, 25 MHz, 30 MHz, 33 MHz, 40 MHz, 50 MHz, 53 MHz, 60 MHz, and 80 MHz, are simulated. Case II-1 is the SSSH case where distances of 50 mm, 150 mm, and 150 mm between S and H are simulated. Case II-2 is the SSMH-SA case where distances of 100 mm, 150 mm, and 200 mm between S and H2 are simulated. Case II-3 is the SSMH-OP case where distances of 50 mm, 100 mm, and 150 mm between S and H2 are simulated.

#### 3.3.3. Cases of Area of the Ground Electrodes (Case III)

When discussing Case III, the frequency of the source is fixed to 20 MHz. The distances of the forward path and the backward path are the same. Three different areas of the ground electrodes, including 50 mm × 50 mm, 75 mm × 75 mm, and 100 mm × 100 mm, are simulated. Case III-1, Case III-2, and Case III-3 are the SSSH case, SSMH-SA case, and SSMH-OP case, respectively, considering three different areas of the ground electrodes.

## 4. Experiment Setup and Results

This section will illustrate the experiment setup and results of BC-WPT. The first subsection shows the implementations of BC-WPT, and the second subsection displays the experiment setup. The measurement and simulation S-parameter model received power with different factors is shown in the third subsection.

### 4.1. Implementations of BC-WPT

**Figure 7:**
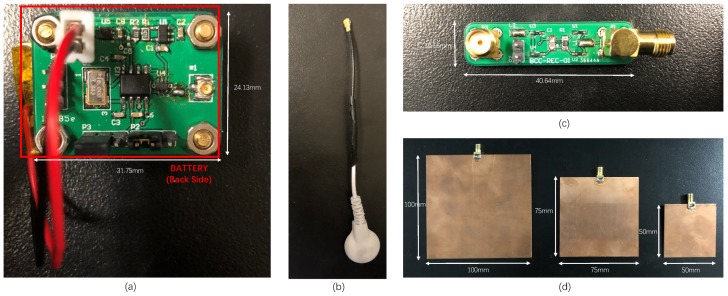
Implementations of BC-WPT. (**a**) Source PCB. (**b**) Signal wire of the source. (**c**) Harvester PCB. (**d**) Ground electrodes with three different areas.

The implementations of BC-WPT are shown in Figure 7. A battery-powered source generator is used, as shown in Figure 7a. The source can generate a sinusoidal wave with the frequency of 20 MHz, 25 MHz, 30 MHz, 33 MHz, 40 MHz, 50 MHz, 53 MHz, 60 MHz, and 80 MHz. The output sinusoidal wave is transmitted to the medical electrode through the IPEX connector. The size of the source PCB is 31.75 mm× 24.13 mm. The signal wire of the source is shown in Figure 7b. The structure of the harvester is the same as that in Figure 5b, and the size of the harvester PCB is 40.64 mm× 10.16 mm, as shown in Figure 7c. Three ground electrodes with different sizes are shown in Figure 7d, including 50 mm× 50 mm, 75 mm× 75 mm, and 100 mm× 100 mm.

### 4.2. Experiment Setup

**Figure 8:**
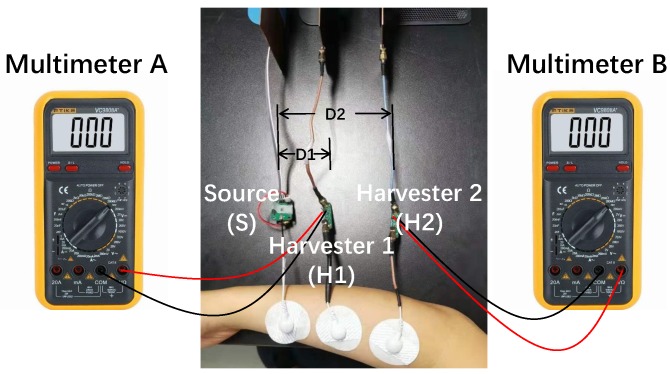
Experiment setup of BC-WPT.

Traditionally, a Vector Network Analyzer (VNA) and Spectrum Analyzer (SA) are commonly used to measure the characteristics of body channel. However, these large bench-top devices may cause building-ground return path and thus affect the measurement results. Therefore, more and more researchers use their own chips or systems for measurement to achieve more accurate measurement results [22,30]. In this paper, the experiment setup of BC-WPT is shown in Figure 8. The experiments are carried out in a general indoor environment. An arm is used for experiments to explore the relationship between received power and different distances between S and H, different frequencies of the source, and different areas of the ground electrodes. The experiment setup is almost the same as the simulation setup. The generated source sinusoidal wave is transmitted to the human body through a medical electrode. A portable multimeter is used to measure the received rectified DC voltage, which is placed far enough from the harvester to avoid building-ground return path. The experiment setup will be calibrated before each measurement, which means Case I-3 at 50 mm distance will be measured and normalized, to offset the impact of varying environmental factors.

### 4.3. Results and Analysis

This subsection discusses the effects of different factors on received power in different cases and the inter-degeneration mechanism. From the following figures, the measurement and simulation results are in good agreement, which illustrates the correctness of the simulated S-parameter model. The simulated circuit model results, which appear in the following figures, will be explained in detail in Section 5.

#### 4.3.1. Cases of Distance Between S and H (Case I)

**Figure 9:**
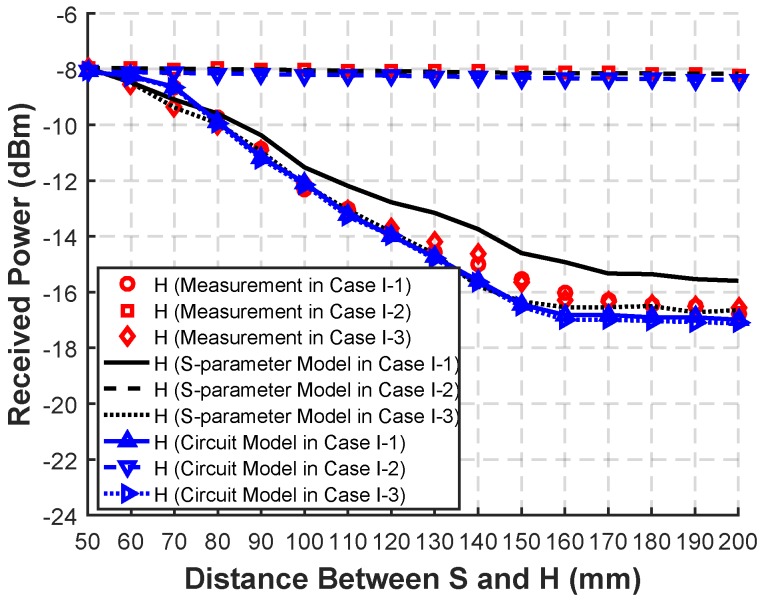
Measurement, simulated S-parameter model, and circuit model received power with different distances between S and H in the SSSH case in Case I-1∼Case I-3, with only backward distance changing in Case I-1, only forward distance changing in Case I-2, and forward and backward distances remaining the same in Case I-3.

In Case I-1∼Case I-3, as shown in Figure 9, it can be seen that the longer the distance between S and H is, the lower the received power is. Especially, the trend in Case I-2 is not obvious at all, which means the forward path has little effect on the results. However, the trend in Case I-1 is more obvious than that in Case I-2, which means the backward path acts more than the forward path. As a result, the trend in Case I-3 is almost the same as that in Case I-1.

**Figure 10:**
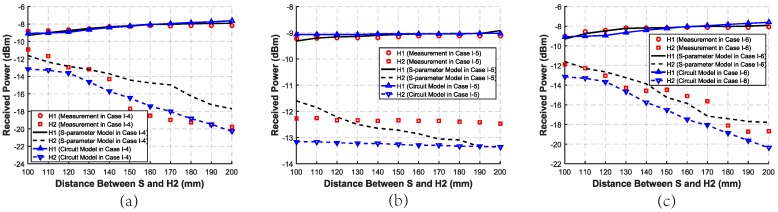
Measurement, simulated S-parameter model, and circuit model received power of two harvesters with different distances between S and H2 in the SSMH-SA case. (**a**) Case I-4 with only backward distance changing. (**b**) Case I-5 with only forward distance changing. (**c**) Case I-6 with forward and backward distances remaining the same.

In Case I-4∼Case I-6, as shown in Figure 10, when the distance between S and H2 increases, the received power of H2 becomes lower as we expect, while that of H1 becomes higher although the position of H1 does not change. When the distance between S and H2 increases, the distance between H1 and H2 also increases. That means when the distance between H1 and H2 increases, the inter-degeneration mechanism on H1 becomes weakly. Especially, the trend in Case I-5 is less obvious than that in Case I-4 and Case I-6, which verifies that the backward path has a greater impact on the results.

**Figure 11:**
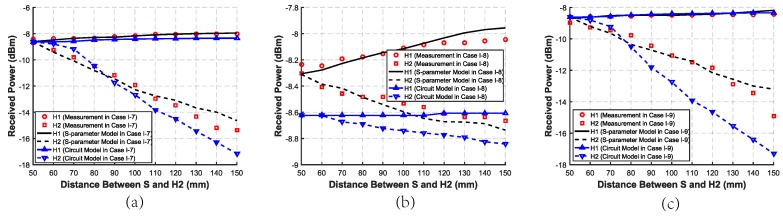
Measurement, simulated S-parameter model, and circuit model received power of two harvesters with different distances between S and H2 in the SSMH-OP case. (**a**) Case I-7 with only backward distance changing. (**b**) Case I-8 with only forward distance changing. (**c**) Case I-9 with forward and backward distances remaining the same.

In Case I-7∼Case I-9, as shown in Figure 11, when the distance between S and H2 increases, the received power of H2 becomes lower, while that of H1 becomes a little higher. That means the distance between H1 and H2 has an inter-degeneration mechanism on both of their received power. As a result, when two harvesters are in a straight line, the inter-degeneration mechanism will become stronger when they get closer to each other. In addition, the backward path has a greater influence on the received power than the forward path in the SSMH-OP case.

#### 4.3.2. Cases of Frequency of the Source (Case II)

**Figure 12:**
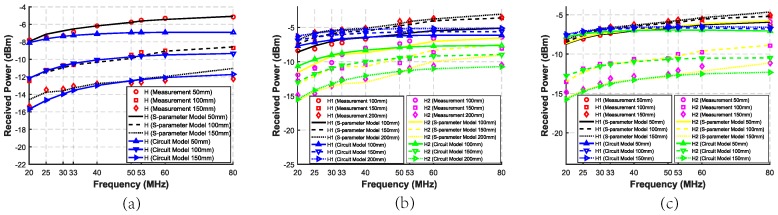
Measurement, simulated S-parameter model, and circuit model received power of two harvesters with different frequencies of the source and different distances where the forward and backward distances remain the same. (**a**) Case II-1: the SSSH case with distances of 50 mm, 100 mm, and 150 mm between S and H. (**b**) Case II-2: the SSMH-SA case with distances of 100 mm, 150 mm, and 200 mm between S and H2. (**c**) Case II-3: the SSMH-OP case with distances of 50 mm, 100 mm, and 150 mm between S and H2.

The measurement and simulation received power in Case II-1∼Case II-3 is shown in Figure 12. When the frequency of the source ranges from 20 MHz to 80 MHz, the received power of H in Case II-1 or both of H1 and H2 in Case II-2 and Case II-3 becomes higher. As the frequency becomes higher, the received power first rises quickly and then tends to become steady, which conforms to the transmission characteristic of the human body channel. As shown in Figure 12, the best transmission frequency of the source is chosen from 40 MHz to 80 MHz.

#### 4.3.3. Cases of Area of the Ground Electrodes (Case III)

**Figure 13:**
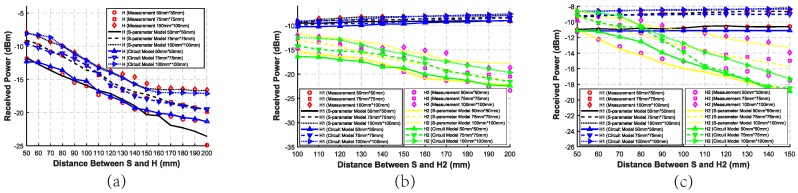
Measurement, simulated S-parameter model, and circuit model received power of two harvesters with different areas of the ground electrodes, including 50 mm × 50 mm, 75 mm × 75 mm and 100 mm × 100 mm, and different distances where the forward and backward distances remain the same. (**a**) Case III-1: the SSSH case with three different areas of the ground electrodes. (**b**) Case III-2: the SSMH-SA case with three different areas of the ground electrodes. (**c**) Case III-3: the SSMH-OP case with three different areas of the ground electrodes.

The measurement and simulation received power in Case III-1∼Case III-3 is shown in Figure 13. As we can see, the larger the area of the ground electrodes is, the higher the received power of H in Case III-1 or both of H1 and H2 in Case III-2 and Case III-3 is. Since the backward path plays an important role in the system, the difference of the areas of the ground electrodes affects the received power obviously. Considering the miniaturization of the source and the harvester, the area of the ground electrodes becomes one of the most important factors.

## 5. Multi-node Modeling of BC-WPT

Based on the simulation parameters in Section 4, an empirical circuit model for multi-node BC-WPT is proposed and an example of the model application is given as follows.

### 5.1. Empirical Circuit Model of Multi-Node BC-WPT

**Figure 14:**
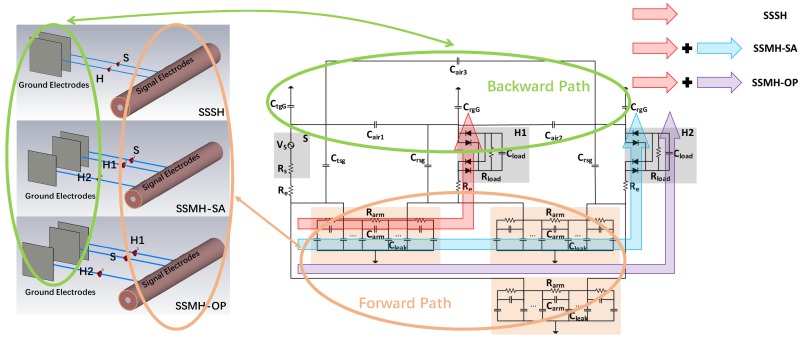
Proposed empirical circuit model and its relationship with the S-parameter model in CST.

The S-parameter model is a black-box model, which cannot reflect the trend of the model with various factors. Therefore, a circuit model is proposed to analyze the variation of the received power with each factor. Based on the simulation parameters of received power, an empirical circuit model can be established, as shown in Figure 14. The relationship between the circuit parameters and the S-parameter model in CST is also shown.

**Figure 15:**
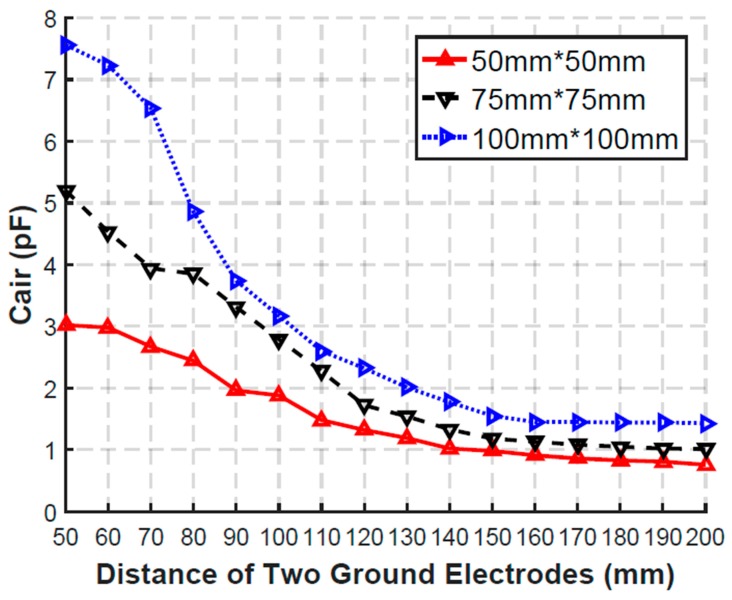
Relationship between $C_{air}$ and distance of two ground electrodes with different areas in CST.

The empirical circuit model consists of the source, the harvesters, the forward path, and the backward path. The source is modeled as the voltage source $V_{s}$ and the source resistor $R_{s}$. The resistor of the electrode and the contact resistor between electrode and skin are denoted by $R_{e}$. The harvester consists of the bridge-structure rectifier and the load of $R_{load}$ and $C_{load}$, which is the same as the experiment setup. The forward path is modeled as an RC network consisting of $R_{arm}$, $C_{arm}$, and $C_{leak}$, where $C_{leak}$ refers to the coupling capacitor between the arm and the ground plane. The backward path consists of two parts. One is the coupling capacitor $C_{air}$ between the floating ground electrodes. The other is the coupling capacitor $C_{tgG}$ and $C_{rgG}$ between the floating ground electrodes of source and harvester and the external ground plane. $C_{tsg}$ refers to the coupling capacitor between the signal and ground electrodes of the source, while $C_{rsg}$ refers to the coupling capacitor between the signal and ground electrodes of the harvester. The parameters of the proposed empirical circuit model are shown in Table 3. The parameters of $R_{arm}$, $C_{arm}$, $C_{leak}$, $C_{tsg}$, $C_{rsg}$, and $C_{air}$ are extracted from CST. The relationship between $C_{air}$ and distance of two ground electrodes is shown in Figure 15.

The circuit model results in Case I, II, and III are shown in Figure 9, Figure 10, Figure 11, Figure 12 and Figure 13, respectively. As can be seen, the circuit model results match the measurement results well, which validates the soundness of the proposed model. The average relative error between the empirical circuit model results and the measurement results in different cases is 12.0%, while that between the S-parameter model results and the measurement results in different cases is 8.7%. As a result, on the basis that the simulation speed of the empirical circuit model is much faster than that of the S-parameter model, the average relative error does not significantly deteriorate, which shows the accuracy and rapidity of the empirical circuit model. The reason for this relative error includes continuous power consumption of the battery during the experiments, which causes the power supply voltage to be unstable, and the contact resistance varying with the degree of tightness between electrodes and the skin.

The cases of two harvesters can be extended to multiple harvesters and the conclusion remains the same. We can conclude from the empirical circuit model that shorter distance between multiple harvesters leads to a stronger inter-degeneration mechanism. And the inter-degeneration mechanism between multiple harvesters is stronger in the case where multiple harvesters are on the same side of the source than that on the opposite side. Besides, suitable frequency and larger area of the ground electrodes can help harvester receive more power.

### 5.2. Application of the Empirical Circuit Model

In this subsection, the guidelines and optimization for application are provided based on the empirical circuit model. Suppose there are two harvesters I and II, the distance between them is 200 mm. The frequency of the source is 20 MHz and the area of the ground electrodes is 100 mm× 100 mm. The question is discussed how to place the source so that the harvesters receive the required power, which is an optimization question [31]. Since the magnitude of the received power varies with the source, the ratio of the received power of two harvesters is considered. In Case I-6 and Case I-9 where the distances of the forward path and the backward path are the same, we can conclude in the simulation platform shown in Table 4. $P_{H1}$ and $P_{H2}$ refer to the simulation received power of H1 and H2. S-H1 and S-H2 refer to the distance between S and H1, and S and H2, respectively. This conclusion gives the sample how to place the source when the position of harvesters is known.

## 6. Conclusions

In this paper, we propose an empirical circuit model to accurately predict the received power of harvesters of a multi-node BC-WPT. The behavior analysis of BC-WPT is performed to provide a theoretical basis. CST and Cadence co-simulation platform is used to extract the parameters of the system. Experiments are also carried out for cross-validation. Three key factors, including distance between the source and the harvester, frequency of the source, and area of the ground electrodes, are taken into consideration, resulting in 15 representative cases to investigate the inter-degeneration mechanism between harvesters when charging multiple nodes simultaneously through BC-WPT. Based on the empirical circuit model, a guideline has been provided for further designs and research on the application of a multi-node BC-WPT system.

## Figures and Tables

**Table 1:** Thickness of Tissue Layers (mm).

	Arm	Leg	Abdomen	Chest
Skin	1.1	1.1	1.1	1.1
Fat	6.1	8.9	1.1	1.1
Muscle	17.3	25.8	7.9	19.4
Cortical Bone	3.8	4.8	-	-
Bone Marrow	4.1	5.2	-	-
Organs	-	-	29.7	18.2

**Table 2:** Simulation Cases of the S-parameter Model.

Case I	Case I-1	The SSSH case with only backward distance changing.
	Case I-2	The SSSH case with only forward distance changing.
	Case I-3	The SSSH case where forward and backward distances remain the same.
	Case I-4	The SSMH-SA case with only backward distance changing.
	Case I-5	The SSMH-SA case with only forward distance changing.
	Case I-6	The SSMH-SA case where forward and backward distances remain the same.
	Case I-7	The SSMH-OP case with only backward distance changing.
	Case I-8	The SSMH-OP case with only forward distance changing.
	Case I-9	The SSMH-OP case where forward and backward distances remain the same.
Case II	Case II-1	The SSSH case with different frequencies.
	Case II-2	The SSMH-SA case with different frequencies.
	Case II-3	The SSMH-OP case with different frequencies.
Case III	Case III-1	The SSSH case with different areas of the ground electrodes.
	Case III-2	The SSMH-SA case with different areas of the ground electrodes.
	Case III-3	The SSMH-OP case with different areas of the ground electrodes.

**Table 3:** Parameters of the Proposed Empirical Circuit Model.

	SSSH	SSMH-SA	SSMH-OP
$V_{s}$	20 MHz–80 M Hz 0 V–3V		
$R_{s}$	50 Ω		
$R_{e}$	100 Ω		
$R_{load}$	2 kΩ		
$C_{load}$	100 pF		
$R_{arm}$ (10 mm)	26.6 Ω		
$C_{arm}$ (10 mm)	1.5 n F		
$C_{leak}$ (10 mm)	0.35 p F		
$C_{air1}$	According to Figure 15		
$C_{air2}$	0 pF	According to Figure 15	0 pF
$C_{air3}$	0 pF	0 pF	According to Figure 15
$C_{tgG}$	1 pF		
$C_{rgG}$	1 pF		
$C_{tsg}$	1.19 p F		
$C_{rsg}$	1.19 p F		

**Table 4:** Simulation Results of the Proposed Empirical Circuit Model.

$\frac{P_{H1}}{P_{H2}}$	Case I-6		Case I-9	
	S-H1 (mm)	S-H2 (mm)	S-H1 (mm)	S-H2 (mm)
1:1	-	-	100.0	100.0
1.5:1	-	-	85.4	114.6
2:1	-	-	72.2	127.8
2.5:1	-	-	59.6	140.4
3:1	-	-	48.4	151.6
4:1	154.3	354.3	-	-
4.5:1	87.6	287.6	-	-
